# Mr. Harrison, on Inoculation

**Published:** 1805-04-01

**Authors:** T. Harrison

**Affiliations:** Kirbymoorside, York


### *To the Editors of the Medical and Physical Journal.*

GENTLEMEN,

FROM the commencement of your widely extended Journal I have read with marked attention every publication which has appeared therein, respecting the cow-pox; and had Mr. Goldson's pamphlet come forth in the early numbers, the cases therein recited would doubtless have increased that scepticism which then arrested me, and which had well nigh resisted those impressive truths, which subsequent experience has constrained me to acknowledge.

Some of your correspondents have regretted that such cases cases ever came before the public; I am, however, disposed to hope, that the avowal of such ambiguous reports will eventually tend to confirm and increase, rather than injure the vaccine practice, a practice founded on the most permanent basis; fixed as the laws of Nature, and irrefragable as truth itself.

It is well known that the propositions of Euclid, and the axioms of our immortal Newton, have been repeatedly carped at; nay, that the truths of the Gospel have been disputed by a modern blasphemous politician, but that those doubts he has attempted to raise in atheistical minds have been happily dissipated by an abler politician, philosopher, and prelate, whose mild answers in his "Apology for the Bible," have abundantly refuted the sophistry of his opponent, and tended in an eminent degree, to establish those prophetic truths he has so ably contended for.

Not more established are these truths, or permanent are those axioms in my estimation, than the principles of vaccination as set forth by our *immortal Jenner*, and confirmed by the suffrage and united testimony of assenting millions.

After such an unqualified avowal, it may be expected that I should bring forward a string of evidence in support of these assertions.—The numbers I have vaccinated, when compared with those of other practitioners, are but inconsiderable; yet some of these, as being accompanied with a peculiarity of circumstances, may not be unworthy of notice.—In November 1800, I vaccinated Master W. aged two years, with recent virus procured from my late much revered friend, Dr. Cappe of York, who at that time laboured greatly to introduce the new practice, and whose important and judicious observations, as to irregularity and anomalous appearances, have been deservedly inserted in your Medical and Physical Journal, (vol. 5, p. 25.) and I may here likewise add, provided Mr. Goldson had attended thereto, it is probable the cases of failure he has given to the world, would have either been suppressed *in toto*, or mentioned only to confirm those truths which have now been above five years on record.—To return to my narrative; my little patient, much to the satisfaction of his friends, passed the disease (if such it may be called) in the most mild, distinct, and unequivocal manner; and in six weeks afterwards, by way of test, was subjected to variolous inoculation, along with a little cousin who had never been inoculated with either vaccine fluid or small-pox matter before. Master W's arms inflamed for a few days at first, and and then subsided; no constitutional affection whatever ensued, whilst his little cousin, inoculated at the same time, and with the same matter, went through the usual routine of disease, had a considerable crop of variolous pustules which maturated kindly, and continued out the usual period; during all which time the two cousins (at my instigation) constantly slept, kissed, and played with each other, but without any effect or consequence whatever.

In December 1800, (as inserted in my letter in the York Herald) the small-pox was very prevalent in this neighbourhood; a subscription was opened for inoculating the poor of this town, and upwards of one hundred presented themselves to a brother practitioner and self. Amongst those which fell to my share, was a mother in her twenty-fifth week of pregnancy, three of her children, under seven years of age, and a sickly unpromising twin of another family, but in the same house. Notwithstanding it was the general wish that variolous matter should be employed, yet from the purest motives of humanity, I could not bring myself to venture thereon, but strongly urged the vaccine fluid, as infinitely milder and better calculated under such precarious circumstances. My recommendation was immediately complied with, the pregnant mother, her three children, and the twins, were immediately vaccinated a few days previous to the general inoculation of the rest with the small-pox. The result was fortunate, the mother and children passed the disease in the most favourable manner, and the former, at the usual period, brought forth a fine healthy girl; the sickly twin, whose life had long been despaired of, from mesenteric obstruction, scarcely suffered any thing from vaccination, and afterwards recovered his general health in a most unexpected manner. These six patients, from unavoidable necessity, mixed with the rest of those subjected to the small-pox inoculation; indeed, the whole atmosphere, from the varied situation of the remaining hundred, might be truly said to be compleatly variolated; and yet, from that moment to the present, they have effectually resisted the small-pox infection.

Allow me now to contrast this case of pregnancy with one less fortunate, which occurred to a medical relative of mine within ten miles of this place.—The patient, a mother (but farther advanced in her pregnancy) was about the same time extremely averse to the cow-pox, and was incessant in her solicitations for variolous inoculation; her medical medical attendant injudiciously consented to her repeated importunities; the consequence was, a premature delivery, the death of the child with the small-pox upon it, and a providential but unexpected recovery in the mother, after a month of undescribable torture and suffering.

It is needless to state that vaccination was with extreme difficulty introduced in this neighbourhood at first, and that the popular clamour against it was strong; fortunately for us in this district, a few in the superior classes of life have subjected their offspring to the new practice, with the happiest success, and those in a lower sphere, perhaps more through fashion than conviction, have been insensibly led on to follow their example. This compliment which the poor have paid the rich, is nothing more than a proper return for the strange mode which the *fashionable nudes* of the present day have so inconsistently introduced, a fashion utterly incompatible with decency, health, and comfort, and which may hereafter draw some animadversions from myself, or an abler pen.

About four years ago, I met with an irregular case of cow-pox in the patient of a neighbouring practitioner; and from a guarded prognosis, at that time, the parents of the young girl were so prudent as to subject her to small-pox inoculation a year afterwards; the event fully verified my fears, and proved her to have been insecure, as she passed the disease in a regular manner, had a few distinct variolous pustules, with a trivial febrile indisposition. Had this case, which put on extensive but spurious inflammation, not been attended to at the proper period, no donbt but it might have lulled the parties into a false security, and have brought the practice into a temporary discredit.

Since the year 1800 to the date of this (February 1805) I have continued to follow the vaccine practice; my patients at various distances have never yet suffered from variolous infection, although they have frequently been exposed to every possible risk, and were at times surrounded with small-pox. Had any failure occurred, averse as the generality of the public at first were, it is but fair to infer they would have sounded the alarm with envious satisfaction. It is true that in some instances I have had to inoculate a second, third, and even a fourth time; yet, this proves only what I have publicly asserted four years ago, viz. "That the cow-pox is assuredly a much milder disease than the small-pox."—" That the insertion of the former is accompanied with more ambiguity than the latter, ter, unless the fluid be employed soon after taking."—"That the failure may be in proportion to its comparative mildness, as its virus, in spite of attentive enclosure, sooner escapes."—"That the utmost circumspection ought to be paid as to its regularity and progress, and that as the disease (like others to which the human constitution is prone) is subjected to certain laws and regulations which are fixed and immutable, it is not less the duty than the interest of every practitioner, sedulously to watch and study these, as emphatically laid down by the great Discoverer, and others, and thereby to discriminate between such true and spurious appearances as may at any future period occur."

During this last month I have vaccinated twenty-two patients without a single failure, with matter obtained from a medical friend at Malton, without any irregular or anomalous appearance; and notwithstanding the many truly laudable and philanthropic Institutes, which are so generally dispersed in the metropolis for the convenience of the medical world, it has been thought expedient by others, as well as myself, to endeavour to establish what I would call a Provincial Jennerian Circle, for the more ready and immediate accommodation of the public with cow-pox virus. On this subject, as well as on others connected therewith, (should this testimony of mine now obtain admittance in your Journal) I may hereafter again address you. At present, I would beg a tender of my thanks to the gentlemen of the different medical committees, who have so generously and publicly stepped forth in distributing gratuitous supplies of vaccine virus to every practitioner in the united kindom.—Their efforts, aided by the concurrence of medical practitioners individually, may, I doubt not, accomplish the *extermination of the small-pox,* and thus free the world from one of the most dreadful maladies that ever raged among mankind.

Although I am professedly a strong advocate for the cow-pox, yet I can hardly subscribe with your worthy correspondent, Mr. Ring, who in his zeal for the good causes, would consign every one inoculating for the small-pox to the utmost rigour of the law.* I would more charitably invite him to give vaccination a fair, candid trial, and then

* Vide Mr. Ring's Compendium of Vaccination at the end of Anstey's Ode to Jenner.

then dare him conscientiously to acquit himself of that trust which a confiding public have placed in him.

It is needless to say that common sense, common honesty, and strict justice, would constrain him, as it has done me, to prefer vaccination, and never to obtrude a severe disease on his patient, when his duty prompts him to recommend a mild one.

I am, &c.

*Kirbymoorside, York, Feb.* 11, 1805. T. HARRISON.

